# Time Release Ion Matrix Regenerates Dystrophic Skeletal Muscle

**DOI:** 10.21203/rs.3.rs-5968078/v1

**Published:** 2025-03-20

**Authors:** Jacob A. Kendra, Alexandra G. Naman, Rebekah L. Blatt, Yava Jones-Hall, Carla D. Zingariello, Richard K. Brow, Steven S. Segal, Aaron B. Morton

**Affiliations:** 1 Department of Kinesiology and Sport Management, Texas A&M University, College Station, TX, 77845; 2 Department of Materials Science & Engineering, Missouri University of Science & Technology, Rolla, MO, 65409; 3 Department of Veterinary Pathobiology, Texas A&M University, College Station, TX, 77845; 4 Department of Pediatrics, University of Florida, 2000 SW Archer Rd., FL, 32610; 5 Department of Medical Pharmacology and Physiology, University of Missouri, Columbia, MO, 65212; 6 Dalton Cardiovascular Research Center, Columbia, MO 65203; 7 Department of Biomedical Sciences, University of Missouri; Columbia, MO 65201; 8 Department of Biomedical, Biological, and Chemical Engineering, University of Missouri; Columbia, MO 65211; 9 Department of Nutrition and Exercise Physiology, University of Missouri, Columbia, MO 65211

**Keywords:** Angiogenesis, Bioactive Glass, CoO-TRIM, Muscular Dystrophy, Regenerative Medicine, Skeletal Muscle, Time Release Ion Matrix, VEGF

## Abstract

A time-release ion matrix (TRIM) restores damaged tissue following injury through local ion release to stimulate regenerative gene expression. Here we report the use of CoO-TRIM, an FDA designated Rare Pediatric Disease Drug, to restore muscle function and structure in the context of debilitating muscle disease. We demonstrate in an established animal model of Duchenne Muscular Dystrophy (DMD), the D2.*mdx* mouse, that tibialis anterior (TA) muscles receiving a single injection of CoO-TRIM exhibit greater active force, myofiber size and regeneration through 70 days post treatment compared to *D2.mdx* receiving vehicle alone. TRIM promoted upregulation of pro-angiogenic growth factor (vascular endothelial growth factor) and increased muscle microvasculature. These findings indicate that CoO-TRIM stimulates growth factors to promote the restoration of muscle structure and function of severely dystrophic mice *in vivo* without toxicity. We conclude that CoO-TRIM is a first-in-class therapeutic compound to combat soft tissue disease by restoring tissue integrity. Moreover, this novel treatment strategy could benefit both early and late stage DMD patients.

## 1. Introduction

Muscular dystrophies (MD) are a group of degenerative skeletal muscle diseases with 40+ genetic mutations, affecting as many as 1:5,000 births worldwide. Many of these mutations encode dysfunctional proteins that diminish myofiber structural integrity, resulting in frequent injury and chronic tissue damage^1–4^. The most common genetic mutation is Duchenne Muscular Dystrophy (DMD), caused by an x-linked mutation of the *DMD* gene and thereby predisposing males^5–9^. As dystrophic muscle undergoes chronic cycles of degeneration and regeneration in response to mechanical strain, cellular repair mechanisms become depleted, culminating in progressive muscle wasting and eventual replacement with non-contractile tissue through increased activity of fibroblasts and adipocytes^10–12^. While a cure for DMD patients remains elusive, advances in multidisciplinary care have slowed disease progression and delayed muscle dysfunction^13^.

Emerging therapies for DMD often utilize gene- and cell- based approaches. Although 8 therapeutics have been approved for DMD, their clinical impact has been questioned by challenges to delivery methods, long-term efficacy, and patient safety^14–16^. The vascular abnormalities (ischemia and dysfunctional dilation) in DMD impair the delivery, and thereby efficacy, of systemic as well as localized gene- and cell- based therapies to affected skeletal muscle by compromising the ability to disperse treatments via the bloodstream^16–20^. Despite developments in these treatment strategies, few effective therapeutic options exist for DMD. This gap in patient care underscores the need for additional approaches for the repair and regeneration of functional muscle tissue.

Impaired angiogenesis and reduced vascular endothelial growth factor (VEGF) contribute to microvascular dysfunction as the pathology of dystrophic muscle develops^21–24^. Moreover, vascular pathology progresses with disease severity. As shown in animal models of DMD, attenuated VEGF expression with reduced microvessel density accompany the increase in capillary-to-myofiber diffusion distance, thereby exacerbating tissue ischemia^18,25,26^. Various methods used to overexpress VEGF such as VEGFA recombinant adeno-associated virus vectors, transplanted *in vivo* muscle-derived stem cells transduced for VEGFA overexpression, removal of miRNA binding sites responsible for inhibiting VEGFA translation, and antibodies binding to VEGFR-1 (VEGF Receptor 1) to increase levels of free VEGF in DMD knock-out (*mdx*) have all increased muscle function, fiber size, capillary density, and tissue perfusion^21,27–29^. In animal models of muscle injury and hindlimb ischemia, VEGF stimulated proliferation and differentiation of myogenic stem cells, which enhanced muscle regeneration while restoring contractile force and preventing apoptosis^30–32^. Such complementary effects of VEGF on angiogenesis and myogenesis suggest VEGF as a potential therapeutic target to combat DMD pathology. However, use of VEGF in the clinic has been unsuccessful due to pathologic angiogenesis in off-target tissues from systemic overdose, while its short half-life (~30 minutes) requires repeated treatments to counteract effects that subside after one day following intravenous infusion^33–35^. These limitations suggest that a novel method for stimulating local production of VEGF to target tissues may be beneficial.

Advances in the design and fabrication of biomaterials have led to novel applications for soft tissue regeneration. Time release ion matrices (TRIMs) are inorganic, non-crystalline, 3^rd^ generation biomaterials, capable of promoting gene expression through controlled temporal release of constituent ions^36–40^. This class of therapeutic has been developed to target bone grafts with injectable cement^41^ or to coat implanted biomedical devices^42^. Historically, the gold standard for TRIM was silica-based (SiO_2_) 45S5 (Bioglass^®^), a bioactive glass with its composition designed to form a hydroxyapatite layer bonding scaffold to bone^43^. However, limitations for 45S5 use in soft tissue include incomplete degradation *in vivo*, as ~85% of silica particles remain 14 days after particle incubation in simulated body fluid ^44,45^. Furthermore, early stages of 45S5 dissolution trigger pH-dependent cytotoxicity: uncontrolled rates of ion exchange in aqueous solutions result in a burst of ion release that alters the pH of local microenvironments^46,47^. Given the limitations of early TRIM compositions based on silicates^48^, borate TRIMs have gained interest due to their rapid degradation and support of cellular proliferation in soft tissue^37,49,50^. In a study investigating borate-based TRIM, micron-sized particles implanted in a rat model of volumetric muscle loss (VML) supported angiogenesis, myofiber regeneration, and VEGF gene expression^51^. Borophosphate-based TRIMs have proven to be ideal candidates for soft tissue (muscle, vasculature, nerves) healing applications due to degradation while maintaining pH neutrality^45,52–54^. However, the ability of a TRIM to stimulate angiogenesis, muscle repair, and regeneration in the context of muscle disease is unexplored.

Here, we demonstrate the use of a borophosphate TRIM to enhance skeletal muscle structure and function in dystrophic D2.*mdx* mice. Our unique composition of CoO-TRIM is formulated to release myogenic and angiogenic ions over time *in vivo* while maintaining a neutral local pH. CoO-TRIM is prepared as a lyophilized powder contained within a vial and resuspended with saline prior to injection within affected myofascial compartments. The present experiments tested two hypotheses: 1) CoO-TRIM enhances muscle force and fiber size in dystrophic tibialis anterior (TA) muscle, and 2) CoO-TRIM promotes angiogenesis. An established mouse model of DMD was used to perform quantitative physiological, histological, and biochemical analyses on the TA muscle to assess the ability of CoO-TRIM to restore structure and function of dystrophic muscle for up to 70 days following a single intramuscular injection.

## 2. Methods

### Biomaterial Fabrication

CoO-TRIM with the nominal composition %wt: 34.6% B_2_O_3_, 35.3% P_2_O_5_, 14.0% CaO, 12.3% Na_2_O, 3.8% CoO was prepared from mixtures of reagent grade calcium carbonate, sodium carbonate, calcium metaphosphate, sodium metaphosphate, boric acid, and cobalt (II) oxide powders using conventional glass processing techniques. Briefly, the raw materials were mixed, placed in a platinum crucible calcined overnight at 300°C to evolve water and CO_2_, then melted at 1150°C for 30 minutes, stirred with a platinum rod, then melted for another 30 minutes. The homogeneous melt was poured into graphite molds, then annealed at 350°C for one hour before cooling to room temperature. Annealed CoO-TRIM was ground to form the CoO-TRIM particles (diameter, <10 microns) using a Spex mill and placed in 1.5 mL microcentrifuge tubes in a desiccator prior to use. Larger particles, in the range of 250–500μm, were prepared by grinding annealed pieces of the CoO-TRIM in a porcelain mortar and pestle, then stored in a desiccator until used.

### Biomaterial Dissolution Kinetics

The dissolution experiments followed those reported for other borophosphate TRIMs^55^. CoO-TRIM particles (300 ± 1 mg; diameter, 250–500 μm) were sealed in a nylon mesh bag with 45 μm openings, then immersed in 50 mL of simulated body fluid (SBF)^55,56^ contained in sealed high-density polyethylene centrifuge tubes immersed in a shaker bath at 37°C. The SBF was prepared as described^56^. Three separate samples were prepared for each timepoint. At the specified timepoints, the bags containing CoO-TRIM were removed, weighed, and their solution pH was recorded at room temperature. Ion concentrations in the dissolution solution were analyzed using Inductively Coupled Plasma – Optical Emission Spectroscopy (ICP-OES) using a Perkin-Elmer Avio 200. The means and standard deviations of the mass loss values, solution pH and ion concentration measurements at each time point are reported. Reacted TRIM particles were dissolved overnight in a solution containing 5 mM EDTA and 0.22 M NaCl prior to analysis by high-performance liquid chromatography (HLPC). HPLC analysis was performed in triplicate with a Dionex GP50–2 pump, an Ionpack AS7 4×250 mm analytical ion exchange column, and an AD25 absorbance detector as described^57^.

### Ethical approval

All protocols in this study were approved by Animal Care and Use Committees at the University of Missouri (AUP 17720) and Texas A&M University (AUP 2022–0215). Animal care was in accordance with the National Research Council’s *Guide for the Care and Use of Laboratory Animals (Eighth Edition, 2011)* and experiments were conducted in accordance with ARRIVE guidelines.

### Animals

Male C57BL/6J mice (RRID: IMSR_JAX:000664, Strain 000664, Jackson Labs; Bar Harbor, ME) and male D2.B10-*DMD*^*mdx*^/J (D2.*mdx*) (RRID: IMSR_JAX:013141, Strain 013141, Jackson Labs; Bar Harbor, ME) mice were purchased from Jackson Labs, housed in the University of Missouri animal care facilities, and studied at 5–7 months of age with age-matched controls. Additional male C57BL/6J mice were housed in the Texas A&M University animal care facilities and studied at 6 months of age for age- and vehicle-matched controls. Male D2.*mdx* mice were selected as the most appropriate mouse model to recapitulate human characteristics of MD pathology in skeletal muscle due to the severity of progressive limb muscle weakness^9^ and X-linked mutation to the *DMD* gene in humans^58^ Mice were housed on a 12h: 12h light: dark cycle at ~23°C, with fresh water and food available *ad libitum*.

### Experiment 1

To validate the use of saline injections as vehicle controls in experiment 2, mice were assigned to 4 experimental groups: 1) untreated C57BL/6J (referred to as Wild Type, WT); 2) WT with saline (vehicle) injection; 3) untreated D2.*mdx*; 4) D2.*mdx* with saline injection to replicate treatment administration. For intramuscular (IM) saline injection (50 μl; 25G needle, BD Biosciences) into the left tibialis anterior (TA) muscle, mice were anesthetized with ketamine and xylazine (100 mg/kg and 10 mg/kg, respectively; intraperitoneal injections), kept warm during recovery, and returned to their cage. Corresponding to 14 days post treatment (dpt), mice were anesthetized (as above), and the left TA muscle was prepared for *in situ* measurements of max isometric force as the criterion for muscle function^59,60^.

### Experiment 2

D2.*mdx* mice were randomly assigned to 2 experimental groups: 1) vehicle alone (*D2.mdx* Saline); 2) CoO-TRIM (*D2.mdx* TRIM). Mice were anesthetized (as above) and 50 μl of saline (*D2.mdx* Saline) or 250 μg of CoO-TRIM suspended in 50 μl of saline (*D2.mdx* TRIM) was injected into the TA muscle of both hindlimbs. A given mouse was studied at one of three time points: 14, 70, and 140 dpt. On the day of an experiment, the left TA muscle was prepared for isometric force measurements as in *Experiment 1*. The mouse was then euthanized via anesthetic overdose and cervical dislocation. TA muscles from both hindlimbs were removed and stored at −80°C for histological and biochemical analyses.

### Muscle Force

The mouse was anesthetized (as above) and weighed. A 2–0 silk suture was secured around the left patellar tendon. The distal tendon of the TA muscle was isolated, secured with 2–0 suture, then severed from its insertion. The mouse was placed prone on a heating platform positioned on a plexiglass board, and the patellar tendon was tied to a vertical metal peg with 2–0 silk suture to immobilize the knee. The TA tendon was tied to a load beam (LCL-113G; Omega, Stamford, CT, USA) coupled to a Transbridge amplifier (TBM-4; World Precision Instruments, Sarasota, FL, USA). The load beam was attached to a micrometer to adjust the optimal length (L_o_), as determined during twitch contractions at 1 Hz^61^. A strip of KimWipe^®^ was wrapped around the exposed TA and irrigated (3 mL/minute) with bicarbonate-buffered physiological salt solution (bbPSS) warmed to 40°C; a heat lamp maintained the muscle at 32°C. The sciatic nerve was isolated, cut proximally, and the distal stump positioned across platinum/iridium (90%/10%) wire electrodes (diameter, 250 μm) for indirect muscle stimulation. Monophasic pulses (0.1 ms, 10 V) were delivered from an S48 stimulator coupled to a SIU5 stimulation isolation unit (Grass Instruments West Warwick, RI, USA). For direct field stimulation, a second pair of wire electrodes was placed across the muscle belly; a Stimu-Splitter II (Med-Lab Instruments, Loveland, CO, USA) was coupled to a second Grass S48 stimulator to generate sufficient current to depolarize myofibers. Maximum tetanic contractions were obtained at 120 Hz for 500 ms. Data for force production were acquired using Power Lab software (ADInstruments, Colorado Springs, CO, USA) on a personal computer.

Following contractions, the TA muscle was excised, blotted of excess moisture, and weighed (XP205 Pro, Mettler Toledo; Columbus, OH, USA). Force production was normalized as N/cm^2^ to account for size differences across muscles; cross-sectional area (CSA) was calculated as: CSA = muscle weight (mg) / [muscle length at L_o_ (mm) × muscle density (1.06 mg/mm^3^)]^62^.

### Immunohistochemistry Analysis

Primary antibodies used were rat anti-CD31 (1:200, RRID: AB_393571, BD Biosciences, Franklin Lakes, NJ, USA), anti-myosin heavy chain 2A (1:50, RRID: AB_2147165, Cat.# SC-71, Developmental Studies Hybridoma Bank, The University of Iowa Department of Biology; Iowa City, IA, USA), and rabbit anti-laminin (1:400, Cat.# NC1732938, Fisher Scientific; Hampton, NJ, USA). Secondary antibodies were all from Fisher Scientific (Hampton, NJ, USA): Alexa Fluor 647 Goat anti-rat (1:400, RRID: AB_2895299, Cat.# A48265), Alexa Fluor 488 Goat anti-mouse (1:133, RRID: AB_2633275, Cat.# PIA32723), Goat anti-rabbit Rhodamine (TRITC) (1:400, RRID: AB_90296, Cat.# AP132RMI).

Left TA muscles were embedded in Tissue-Plus^™^ O.C.T. Compound (Scigen, Fischer Scientific, Hampton, NJ, USA), frozen in liquid nitrogen-cooled 2-methyl butane (Thermo Fisher Scientific, Waltham, MA, USA) and sectioned (thickness, 10 μm) in a Cryostar NX50 Cryostat (Epredia, Kalamazoo, MI, USA) at −17°C onto a microscope slide. Sections were fixed with ice-cold 4% paraformaldehyde for 20 minutes, washed 3x in Tris-buffered saline (TBS), then permeabilized in 0.5% Triton X-100. Primary antibodies were incubated for 60 minutes at room temperature (RT) in blocking buffer (Pierce^™^ Protein-Free Blocking Buffer, Thermo Fisher Scientific; Waltham, MA, USA). Sections were washed 3x in TBS, incubated with secondary antibodies in blocking buffer for 60 minutes at RT, washed 3x in TBS, and mounted in Invitrogen^™^ ProLong^™^ Gold antifade reagent with DAPI (Cat.# P36941, Fisher Scientific, Hampton, NJ, USA). Slides were imaged on a Stellaris 5 White Light Laser confocal microscope (Leica Microsystems, Deer Park, IL, USA) using Leica LAS_X software (RRID: SCR_013673, Leica Microsystems, Deer Park, IL, USA).

For each muscle cross section, 3–5 randomized 580 × 580 μm regions of interest were imaged with 10x (N.A., 0.40) or 20x (N.A., 0.75) objectives and values across all regions were averaged per muscle. At least 300 myofibers per TA muscle section were evaluated. Fiber CSA was analyzed via semi-automatic muscle segmentation analysis in *NIS*-Elements Advanced Research software (RRID: SCR_014329, Nikon, Melville, NY, USA) presented as μm^2^. Using NIH ImageJ (RRID: SCR_003070, NIH, Bethesda, MD, USA), the number of fibers with central nuclei were counted and compared to the total number of fibers as an index of myofiber regeneration. The total area of CD31^+^ staining per fiber was measured to evaluate microvascular area using Aivia machine-learning software (Leica LAS_X Software imported calibration: 0.56 μm/px) (Aivia, Bellevue, WA, USA).

### Confocal imaging and vascular analysis

At 14 dpt, a *D2.mdx* TRIM and *D2.mdx* Saline mouse was anaesthetized (as above), and wheat germ agglutinin conjugated to Alexa Fluor 647 (WGA 647; #W32466, Thermo Fisher) was injected (200 μl; 1 mg/mL) into the retroorbital sinus, allowed to circulate for ~10 minutes to stain the endothelial glycocalyx, then skin and overlying connective tissue were removed to expose the gluteus maximus (GM) muscle^61,63–65^. While viewing through a stereomicroscope, the muscle was irrigated with 0.9% sterile saline, excised, and placed in a 12-well plate coated with transparent silicone elastomer (Sylgard 184^™^ Midland, MI, USA) that contained chilled (4°C) phosphate-buffered saline (PBS; pH 7.4; #P3813, Sigma). Insect pins secured the muscle at its edges to approximate in situ dimensions with the ventral surface facing up to optimize visualization of the microvasculature. Excessive connective tissue and fat were removed using microdissection. After rinsing in PBS, the muscle was immersed in 50%, 75%, and 100% MeOH, each for 5 minutes The muscle was cleared in Visikol^®^ HISTO-1^™^ and Visikol^®^ HISTO-2^™^ clearing solution (Sigma) per manufacturer’s instructions and then imaged.

A cleared GM muscle was placed in an imaging chamber with the ventral surface facing the objective to optimize resolution of the WGA-stained microvasculature. To create a smooth imaging plane devoid of air bubbles with the same refractive index, ~10 μL of Visikol^®^ HISTO-2^™^ was added to the chamber, and the tissue was flattened by placing a glass block (2.5 cm × 2 cm × 1 cm; mass, 7.8 g) on the dorsal surface. Images were acquired using a 10x objective (N.A., 0.40) on a confocal microscope as above^64^.

### Evaluation of Toxicity

Following euthanasia, kidney, liver, lung, and heart were removed (n=3) from both groups in the 14-day timepoint and frozen in O.C.T (as above). Toxicity was evaluated morphometrically for signs of pathology in tissue cross sections by a board-certified veterinary pathologist in the College of Veterinary Medicine & Biomedical Sciences Core Histology Laboratory (RRID: SCR_022201, Texas A&M University, College Station, TX).

### Muscle Tissue Preparation

Right TA muscle samples from *D2.mdx* Saline and *D2.mdx* TRIM mice at each timepoint were homogenized in lysis buffer (pH = 7.4) containing 5 mM Tris-HCI, 5 mM EDTA with protease and phosphatase inhibitors (Sigma-Aldrich, St. Louis, MO, USA). Homogenates were centrifuged at 1,500 *g* for 10 minutes at 4 °C. The supernatant (soluble fraction) was aspirated, and its protein concentration assessed using the Bradford method (Sigma-Aldrich, St. Louis, MO, USA).

### Western Blot Analysis

Protein concentration of each sample was normalized in 4x Laemmli sample buffer (Cat. # 1610747, Bio-Rad, Hercules, CA, USA) containing 5% dithiothreitol. Samples were loaded on 4–20% gradient Criterion TGX Midi-Protein gels (Bio-Rad, Hercules, CA, USA) for electrophoresis and transferred to LF-PVDF membranes (Millipore, Burlington, MA, USA). Following transfer, membranes were blocked in either 5% milk solution or Intercept TBS Blocking Buffer (Li-Cor Biotechnology, Lincoln, NE, USA) for 1 h at room temperature; followed by incubation with primary antibodies overnight at 4 °C. Membranes were exposed to either IRDye 800CW Goat anti-Mouse IgG (RRID: AB_621842, Cat.# 926–32210) or IRDye 800CW Goat anti-Rabbit IgG secondary antibodies (RRID: AB_621843, Cat.# 926–32211) (Li-Cor Biotechnology, Lincoln, NE, USA) for 30 – 60 minutes depending on the protein of interest. All primary and secondary antibodies use a 1:1000 and 1:20,000 concentration, respectively, unless otherwise noted. Membranes were scanned with a Li-Cor Odyssey DLx Imager (Li-Cor Biotechnology, Lincoln, NE, US) and analyzed using Image Studio Lite. Primary antibodies of interest were; phospho-Akt (Ser473) (RRID: AB_329825, Cat.# 9271; Cell Signaling Technology, Danvers, MA), Akt1 (RRID: AB_915788, Cat.# 2938; Cell Signaling Technology, Danvers, MA), Phosphor-mTOR (Ser2448) (RRID: AB_10691552, Cat.# 5536; Cell Signaling Technology, Danvers, MA), mTOR (RRID: AB_2105622, Cat.# 2983; Cell Signaling Technology, Danvers, MA), phospho-4E-BP1 (Thr37/46) (RRID: AB_560835, Cat.# 2855; Cell Signaling Technology, Danvers, MA), 4eBP1 (RRID: AB_2097841, Cat.# 9644; Cell Signaling Technology, Danvers, MA), Alpha II spectrin (RRID: AB_2194351, Cat.# sc-48382; Santa Cruz Biotechnology, Santa Cruz, CA, 1:250 incubated overnight at 4 °C then 3 h at room temperature secondary concentration 1:5,000), LC3B (RRID: AB_915950, Cat.# 2775; Cell Signaling Technology, Danvers, MA, secondary concentration 1:5,000), Phospho-STAT3 (Tyr705) (RRID: AB_2491009, Cat.# 9145; Cell Signaling Technology, Danvers, MA), STAT3 (RRID: AB_331269, Cat.# 4904; Cell Signaling Technology, Danvers, MA). Western blots were normalized to total protein according to recommendations for fluorescent Western Blotting^66^ using Revert total protein stain (Cat.# 926–11011, Li-Cor Biotechnology, Lincoln, NE, USA). The 40 kDa bands correlate with the total protein in each lane and are shown as a representative image of the protein loading. Individual data points are displayed as percentages of corresponding *D2.mdx* Saline means. Full images of analyzed blots and corresponding total protein are displayed in supplementary figure 5 of the data supplement.

### ELISA

Samples of muscle supernatant (50 μL; referred to above) were analyzed to determine VEGFA protein concentration using a Quantikine enzyme-linked immunosorbent assay (ELISA) (Cat.# MMV00, R&D Systems, Minneapolis, MN, USA) according to the manufacturer’s instructions. Concentrations were determined by comparing samples to recombinant VEGFA protein standard supplied by the manufacturer.

### Statistical Analysis

Summary data are reported as means ± S.E.M. Statistical analyses were performed using Prism 9 software (RRID: SCR_002798, GraphPad Software, La Jolla, CA, USA). One-way analysis of variance (ANOVA) and Student’s *t-test* were used, when appropriate, to determine statistical significance among group mean differences. Values for “*n*” refer to the number of mice studied in each experimental group. *P* ≤ 0.05 was considered statistically significant.

## 3. Results

### TRIM Properties

**Figure 1.**
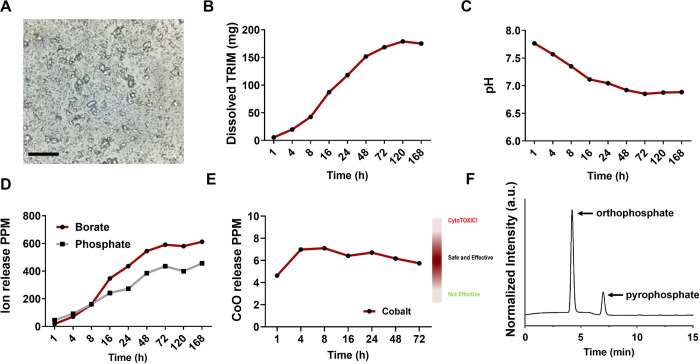
Dissolution kinetics of TRIM. **A)** Representative image of TRIM particles suspended in simulated body fluid (SBF). **B)** Weight release of TRIM particles (300 mg) in 50 mL of SBF across 7 days (168 h). **C)** TRIM dissolved in SBF maintains neutral pH across 7 days. **D)** Release kinetics of Borate and Phosphate ions. **E)** Cobalt ion release profile in a safe and effective range across 72 h. **F)** High-performance liquid chromatography (HPLC) chromatograph of TRIM after reaction in SBF for 7 days, revealing the formation of an amorphous calcium polyphosphate (ACpP) phase composed of orthophosphate and pyrophosphate anions. Scale bars = 10 μm

Figure 1 summarizes weight dissolution, changes in pH, and ion release from TRIM particles immersed in SBF at 37°C across the indicated time points. Like prior TRIMs that are known for rapid degradation (*i.e.*, days) and conversion to hydroxyapatite (HA)^46^, the initial release characteristics of the CoO-TRIM occur over the first 2–3 days, dissociating 58% of its mass after one week in SBF (Figure 1B). Addition of phosphate to the CoO-TRIM composition fostered a pH-neutral environment in contrast to the typical, alkaline TRIMs^55^ (Figure 1C). Ion release kinetics for borate and phosphate revealed their congruent release across one week in SBF (Figure 1D) at a range capable of stimulating regeneration and angiogenesis^39,67^. Cobalt, while cytotoxic in excess, is a known stimulator of angiogenesis^39,68,69^ and inhibits HA formation when used in a BG^70^. CoO-TRIM provides a consistent, physiologically safe and effective range of cobalt release (Figure 1E) over 3 days. Consistent with an earlier study of a borophosphate TRIM^55^, after one week in SBF, HPLC chromatograph demonstrate CoO-TRIM particles also convert to an amorphous calcium polyphosphate (ACpP) phase that includes orthophosphate and pyrophosphate anions (Figure 1F). The chromatograph indicates that about 12% of the P-anions in the ACpP phase are associated with pyrophosphate anions which are known to inhibit HA formation^71^.

### Experiment 1

#### Vehicle administration alone does not enhance muscle function

**Figure 2.**
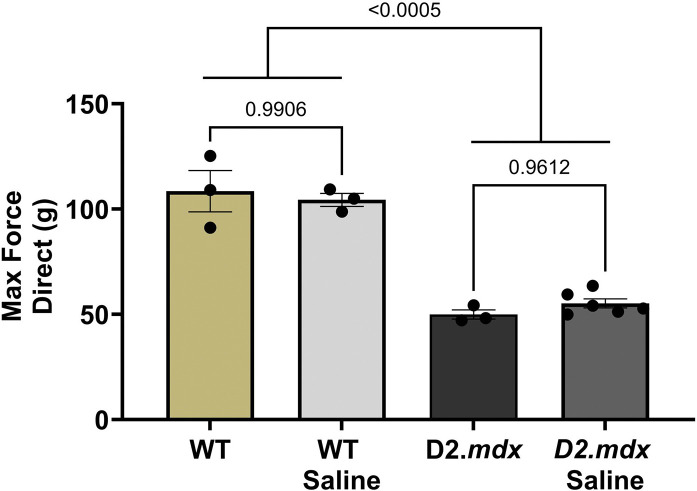
Validation of saline injection as vehicle controls in experiment 2. Summary values of max TA forces from WT, WT Saline, D2.*mdx*, and D2.*mdx* Saline mice (n= 3–6) at 14 dpt; Summary values are means ± SEM, p<0.05 = significant.

During direct stimulation at 120 Hz, max tetanic force developed by the TA muscle was not different between WT and WT with saline injection (Figure 2). In D2.*mdx* mice, max TA muscle force was reduced by ~half vs. WT mice (Figure 2). At 14 days post saline injection, TA muscle in D2.*mdx* mice was reduced by a similar amount (Figure 2), with no effect of vehicle alone.

### Experiment 2

#### TRIM administration is not toxic

Although D2.*mdx* mice develop epicardial calcification with disease progression^72^, there was no overt pathology in the lung, kidney or liver tissue samples (n=3) at 14 dpt. Nor were differences observed in the appearance of these tissues in cross-section from *D2.mdx* TRIM mice when compared to *D2.mdx* Saline.

#### TRIM Treatment Enhances TA Muscle Force

**Figure 3.**
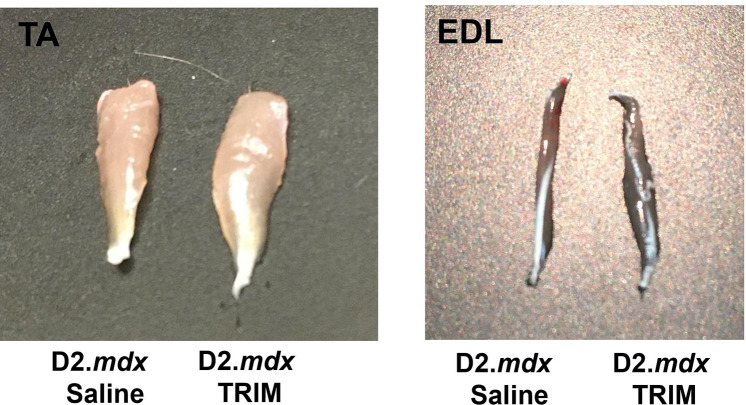

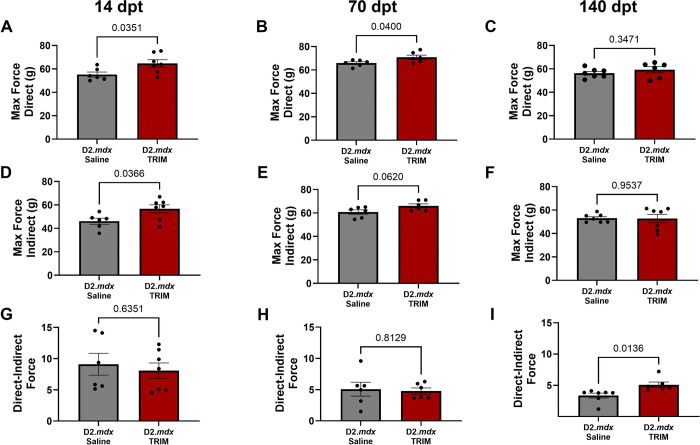
TRIM treated muscles are visibly larger, and exhibit enhanced max force of hindlimb muscles from D2.*mdx* mice. **3.1,** Representative images of TA and extensor digitorum longus (EDL) muscles from D2.*mdx* mice without and with TRIM at 14 dpt. Dystrophic muscles are visibly larger with TRIM. **3.2, A-C)** Top: Summary values for max absolute force via direct muscle stimulation at 120 hz **A)** at 14 dpt, **B)** 70 dpt, and **C)** 140 dpt. **D-F)** Middle: max absolute force via indirect stimulation at 120 hz **D)** at 14 dpt, **E)** 70 dpt, and **F)** 140 dpt. **G-I)** Bottom: Neuromuscular difference, calculated as the difference between absolute force during direct vs. indirect stimulation at **G)** 14 dpt, **H)** 70 dpt, and **I)** 140 dpt. (n=6–7); Summary values are means ± SEM, p<0.05 = significant.

At 14 and 70 dpt, there were no significant differences in body weight between *D2.mdx* Saline and *D2.mdx* TRIM mice (Table 1). However, at 140 dpt, body weight was greater in TRIM mice (P= 0.015). TA muscle weight trended higher for TRIM treated compared to *D2.mdx* Saline mice across all time points (Table 1) consistent with observed slight increase in TA and extensor digitorum longus (EDL) size (Figure 3.1). At 14 and 70 dpt, TA absolute muscle force (g) in D2.*mdx* mice improved with TRIM (17%, P=0.035 and 8%, P=0.04, respectively) with direct stimulation (Figure 3.2A and 3.2B). With indirect excitation through the sciatic nerve, force (g) was 23% (P=0.037) and 8% (P=0.062) greater at 14 and 70 dpt with *D2.mdx* TRIM vs. *D2.mdx* Saline (Figure 3.2D and 3.2E). There was no difference in either direct or indirect force between groups at 140 dpt (Figure 3.2C & 3.2F). Accounting for differences in muscle cross-sectional area, there were no differences in specific force (N/cm^2^) using either direct or indirect stimulation (Supplementary Figure 2). The difference between max force values obtained with direct vs. indirect stimulation was taken to evaluate differences in neuromuscular function^73^, with TRIM treated being greater than D2.*mdx* saline mice at 140 dpt (Figure 3I).

#### Regenerative Capacity is Restored in TRIM TA Muscle

**Figure 4.**
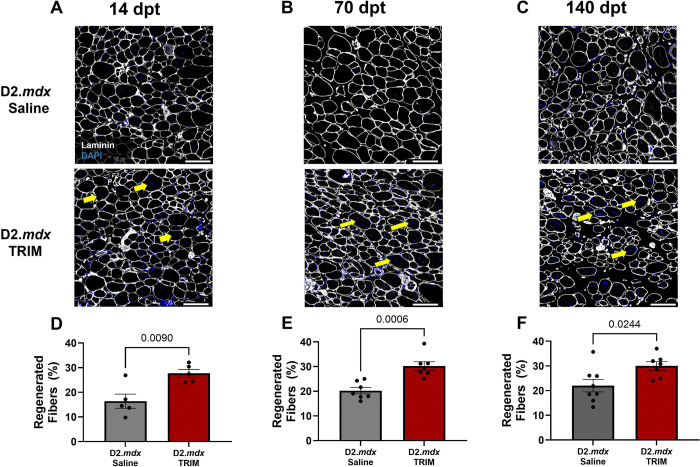
Comparison of centrally located nuclei in muscle fibers of *D2.mdx* Saline and TRIM mice. **A-C)** Representative images of TA muscle cross sections at 14 dpt, 70 dpt, and 140 dpt. Laminin (white): basal laminae; DAPI (blue): nuclei. **D-F)** Summary values for % CNFs, calculated as: (centrally nucleated fiber #/total fiber #) × 100 in randomized regions of interest. Summary values presented for **D)** 14 dpt, **E)** 70 dpt, and **F)** 140 dpt. (n=5–8); Summary values are means ± SEM, p<0.05 = significant. Scale bars = 100 μm

To determine the effect of TRIM on muscle regeneration, nuclei were counterstained with DAPI, and centrally nucleated fibers (CNF) were compared to the total number of myofibers within respective ROIs. TRIM-treatment of D2.*mdx* mice increased myofiber regeneration by 11% at 14 dpt over *D2.mdx* Saline mice (Figure 4A and 4D). Furthermore, TRIM promoted a 10% and 8% increase in regenerated fibers vs. D2.*mdx* Saline mice at 70- and 140 dpt, respectively (Figure 4B and 4E; Figure 4C and 4F), suggesting that TRIM restores the regenerative capacity of dystrophic myofibers in D2.*mdx* mice^12^.

#### Myofiber CSA is Increased in TRIM-Treated TA muscles

**Figure 5.**
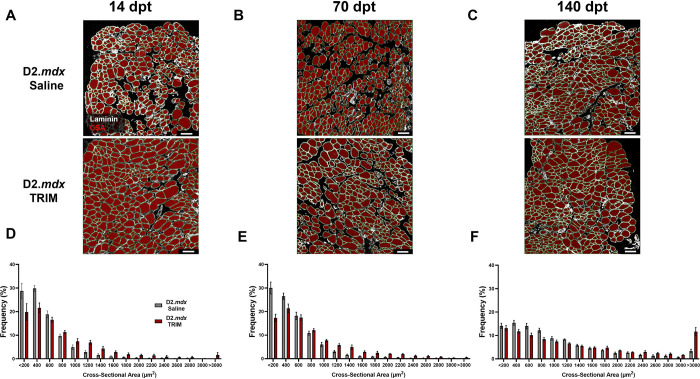
TRIM increases dystrophic TA muscle fiber size. **A-C)** Representative images of TA muscle cross-sections at **A)** 14 dpt, **B)** 70 dpt, and **C)** 140 dpt. Laminin (white) identified basal laminae surrounding myofibers (red), which were quantified for CSA. **D-F)** Muscle fiber CSA distribution at criterion timepoints. Summary values are means ± SEM. Scale bars = 100 μm

**Figure 6.**
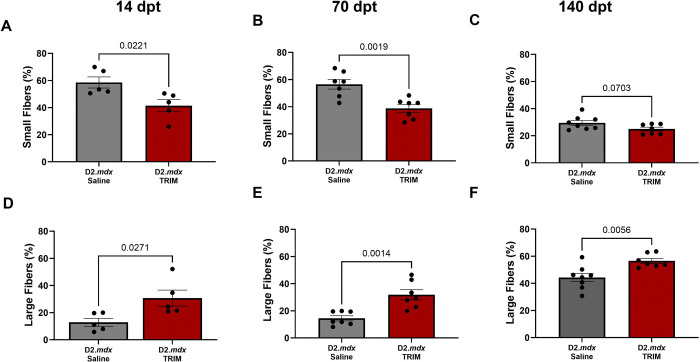
TRIM increases frequency (%) of large fibers up to 140 dpt and reduces small fibers up to 70 dpt. **A-C)** Summary values comparing small fiber frequency at **A)** 14 dpt, **H)** 70 dpt, and **I)** 140 dpt. **D-F)** Summary values comparing large fiber frequency at **D)** 14 dpt **E)** 70 dpt **F)** 140 dpt. (n=5–8); Summary values are means ± SEM, p<0.05 = significant.

Myofiber CSA was partitioned into 200 μm^2^ increments to create relative frequency histogram plots for *D2.mdx* Saline and *D2.mdx* TRIM groups. At both 14 and 70 dpt, TRIM shifted the CSA frequency curves to the right (Figure 5A–B and D–E) due to an ~18% reduction in small fibers (<400 μm^2^) and an ~18% increase in large fibers (>1000 μm^2^) (Figure 6A–B and D–E). In contrast, at 140 dpt, TRIM no longer shifted the CSA frequency curve to the right (Figure 5C and F) nor reduced the number of small fibers (Figure 6C), yet there was still a 12% increase in larger fibers compared to the *D2.mdx* Saline group (Figure 6F).

#### Muscle Protein homeostasis shifts with TRIM treatment

**Figure 7.**
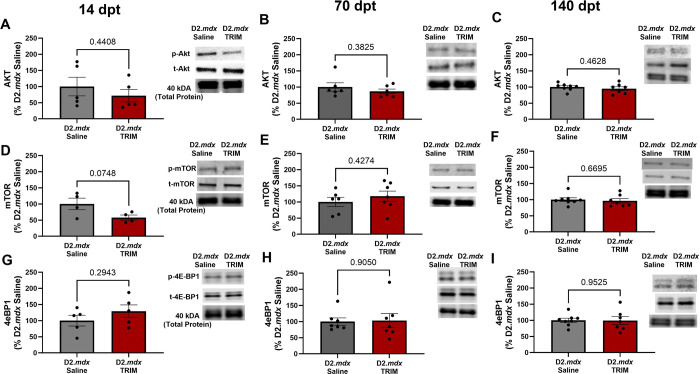
Anabolic signaling unaffected by TRIM. (Top) **A-C)** Representative immunoblots and mean densitometric data for ratio of phosphorylated Akt to total Akt in *D2.mdx* Saline and *D2.mdx* TRIM TA muscle at **A)** 14 dpt, **B)** 70 dpt, and **C)** 140 dpt. **D-F)** Phosphorylated mTOR to total mTOR immunoblots and accompanying densitometric data (middle) for **D)** 14 dpt, **E)** 70 dpt, and **F)** 140 dpt. Downstream target for protein synthesis translation, 4E-BP1, representative immunoblots and summary densitometric values for **G)** 14 dpt, **H)** 70 dpt, and **I)** 140 dpt. Blots were normalized to total protein stain reflected by 40 kDa band. (n=5–8); Summary values are means ± SEM, p<0.05 = significant. Blots were cropped for clarity. Full images available in supplementary figure 5.

Given the increases in muscle force and myofiber CSA in response to TRIM, we immunoblotted for phosphorylated Akt, mTOR, and 4E-BP1 to investigate whether TRIM promoted anabolic signaling. Across all time points, there was no statistical difference in Akt/mTOR pathway activation between groups (Figure 7 A–I). These data suggest that the increases in TRIM-induced muscle force and fiber size are not attributed to increases in Akt/mTOR activation.

**Figure 8.**
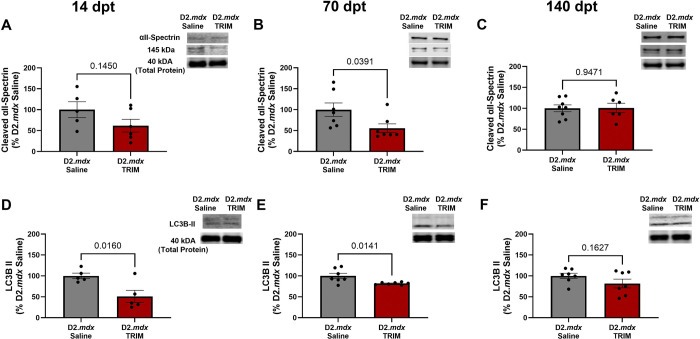
TRIM modulates proteolytic activity. **A-C)** Representative immunoblots (top) for αII-Spectrin at 240 kDA and its cleavage product at 145 kDa were normalized to total protein represented by the 40 kDa band. Mean densitometric data (bottom) revealed a downward trend of cleaved αII-Spectrin abundance in TRIM mice at 14 dpt, with a significant reduction for the TRIM group at 70 dpt. There was no difference at 140 dpt. **D-F)** Representative immunoblots (bottom) of LC3B II and mean densitometric data (bottom) at **A)** 14 dpt and **B)** 70 dpt, revealed TRIM reduced autophagosome number (p=0.02) and (p=0.01) respectively. No differences were observed at **C)** 140 dpt. (n=5–8); Summary values are means ± SEM, p<0.05 = significant. Blots were cropped for clarity. Full images available in supplementary figure 5.

Calpain activation is a known contributor to the proteolysis of dystrophic muscle^74–76^. Active calpain cleaves the skeletal muscle structural protein αII-spectrin from 240 to a 145 kDa product. Therefore, we measured calpain activity by western immunoblotting for αII-spectrin 145 kDa cleaved protein byproduct. TRIM treatment trended at 14 dpt and reduced calpain activation at 70 dpt, vs. Saline, (P= 0.14) and (P=0.03) respectively (Figure 8A and B). However, by 140 dpt, there was no difference between groups (Figure 8C). The autophagy marker^77^ LC3BII was reduced in TRIM mice at 14 and 70 dpt, (P=0.02) and (P=0.01) while not significant at 140 dpt (P=0.16) (Figure 8D–F) compared to D2.*mdx* Saline mice. Taken together, these results suggest TRIM can reduce Ca^2+^-dependent proteolytic activity and autophagosome abundance.

#### TRIM Enhances Angiogenesis

Ions released by CoO-TRIM (borate, phosphate, and cobalt) promote angiogenesis^38,39,51,78^. Imaging revealed a robust capillary network following TRIM treatment with greater depth of perfusion (Figure 9.1). In muscle cross sections we evaluated the area of CD31, a well-defined biomarker of endothelial cells that increases with vessel growth^79,80^. This analysis revealed a >2-fold increase in EC area in TA muscles of TRIM mice at all three timepoints compared to *D2.mdx* Saline (Figure 9.2D–F), indicating that TRIM promotes angiogenesis in MDs, consistent with the effects of other TRIM compounds applied to soft tissue injuries^36,39,51^.

#### VEGF is Increased in D2.mdx Mice Following TRIM Treatment

**Figure 10.**
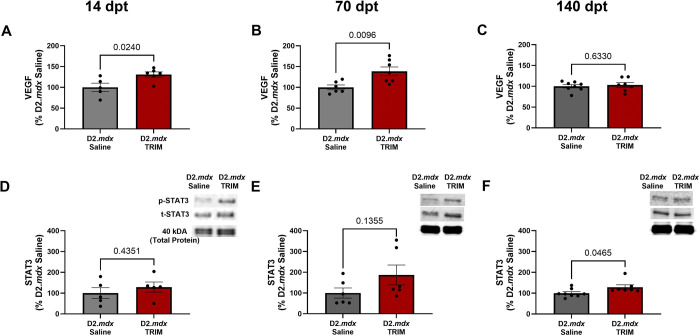
Angiogenic growth factors are increased with TRIM. **A-C)** VEGF concentration in whole muscle TA homogenates probed for by ELISA at **A)** 14 dpt, **B)** 70 dpt, and **C)** 140 dpt. **D-F)** Representative immunoblots (top) of the ratio of phosphorylated STAT3 to total STAT3 and mean densitometric data (bottom) at **D)** 14 dpt, **E)** 70 dpt, and **F)** 140 dpt. (n=5–8); Summary values are means ± SEM, p<0.05 = significant. Blots were cropped for clarity. Full images available in supplementary figure 5.

VEGF is a potent mediator of angiogenesis^81–83^, while signal transducer and activator of transcription 3 (STAT3) is a regulator of VEGF expression^84,85^. Previous studies have revealed improvements in dystrophic muscle function with increased VEGF expression^21,27–29^. At 14 dpt, VEGF concentration was 31% greater (P=0.02) following TRIM treatment (Figure 10A) and remained elevated at 70 dpt (P=0.01) vs. *D2.mdx* Saline (Figure 10B). At 140 dpt, there was no longer any difference between groups (Figure 10C). These data coinciding with the increases seen in muscle force (Figures 3A and 3B), myofiber CSA (Figures 5D and 5E) and reductions in autophagosome abundance (Figures 8D and 8E) at 14- and 70 dpt with TRIM treatment are consistent with a VEGF-mediated contribution to muscle function.

At 14 dpt, STAT3 phosphorylation was 30% greater in *D2.mdx* TRIM vs. *D2.mdx* Saline (P=0.44) with a similar trend at 70 dpt (P=0.14) (Figure 10D and E). At 140 dpt, TRIM increased STAT3 phosphorylation vs. *D2.mdx* Saline (P=0.04) (Figure 10F), suggesting that STAT3 may contribute to the maintenance of microvessels reestablished during the angiogenic response.

## 4. Discussion

Borate ion matrices injected as micron-sized particles are promising TRIMs, restoring damaged skeletal muscle, stimulating regenerative gene transcription through local ion release following injury^38,51^. The formulation of TRIM used in the present experiments was designed to release biologically active concentrations of borate, phosphate, and cobalt ions, promoting skeletal muscle regeneration and angiogenesis to enhance muscle function. While a previous study investigated the effect of a TRIM in the context of volumetric muscle loss^51^, none have investigated its application to skeletal muscle disease. To gain this insight, we tested the hypothesis that CoO-TRIM in dystrophic muscle would 1) enhance muscle force and fiber size, and 2) promote angiogenesis. The TA muscle in the mouse hindlimb was studied owing to its clinical relevance in DMD, its superficial location, and accessibility for isometric force measurements^59,86^. Following a single intramuscular injection of CoO-TRIM, key findings from these experiments are: 1) Local delivery is not toxic to other organs, 2) Max isometric force was enhanced up to 70 dpt; 3) regenerative capacity in dystrophic muscle was improved; 4) myofiber size increased compared to *D2.mdx* Saline; 5) proteolytic markers were reduced up to 70 dpt; 6) angiogenesis was enhanced up to 140 dpt; 7) VEGF protein was enhanced up to 70 dpt.

### Properties of TRIMs during restoration of skeletal muscle

Silicate TRIMs have been studied for their bone regeneration and wound healing properties^37,49,68,87^, with new applications to skeletal muscle^51^. The use of borate TRIMs has increased due to their rapid degradation and increased bioactivity as compared to traditional silica-based TRIMs (Bioglass^®^, calcium silicates) ^37,39,44,46^. Boron plays an essential role in angiogenesis and regeneration^50,88^ but its rapid release from borate TRIMs can promote alkalization and toxicity^89^. To avoid these effects, glass compositions can be modified to control degradation and pH-neutrality^49,55,78^. For example, inclusion of dopant ions (PO_4_^3−^, Cu^2+^, Al^3+^, Co^2+^, etc.) serves to strengthen the matrix structure and regulate degradation to provide a therapeutic environment for regeneration, thereby enhancing their long-term biological actions^39,51,67,90,91^. For example, borophosphate-based ion matrix kinetics as developed for these experiments have controlled degradation and solubility for soft tissue repair^46,52,55,78^, while enhancing muscle regeneration and formation of prototypic myofibers^54^. The present findings demonstrate that our novel CoO-TRIM composition sustains gradual release of ions in a therapeutic range, stimulating regeneration while maintaining a pH-neutral environment.

TRIMs are typically designed to form hydroxyapatite (HA) layers upon implantation within biological environments during dissolution^46^. While beneficial for bone, HA formation can promote myofiber calcification (i.e. myositis ossificans) making traditional TRIMs undesirable for soft tissue applications. HA forms under basic conditions while pH-neutral TRIMs (as used here) inhibit HA due to the formation of an amorphous calcium polyphosphate (ACpP) layer containing orthophosphate and pyrophosphate anions^55,78^, which inhibit HA formation^71^. Furthermore, incorporation of cobalt ions inhibits HA formation^70,87^. The present study demonstrates the capabilities of CoO-TRIM to release cobalt in a safe yet effective range (Figure 1E) while degrading into an ACpP material containing pyrophosphates (Figure 1F and Supplementary Figure 1). From these results, we suggest CoO-TRIM controls ion release when injected into dystrophic skeletal muscle, maintains pH-neutrality, and inhibits HA formation while stimulating myofiber regeneration and angiogenesis, thereby reversing the adverse outcomes of MD.

### Skeletal muscle force following treatment with CoO-TRIM

The D2.*mdx* mouse exhibits severe skeletal muscle pathology characterized by progressive atrophy and weakness, recapitulating a severe DMD phenotype^9^. At 6 months of age, D2.*mdx* hindlimb muscles present with severe atrophy and a 50% reduction of muscle strength^92^. Chronic myofiber degeneration exhausts the regenerative capacity of dystrophic skeletal muscle culminating in satellite cell depletion^11,12,93,94^, tissue necrosis and impaired muscle function^10,95,96^. While borate TRIMs have repeatedly demonstrated positive effects on soft tissue regeneration^39,51,97^, this is the first study to highlight the ability for a TRIM to promote functional recovery of diseased skeletal muscle. Herein, we report that CoO-TRIM enhances the regenerative response of skeletal muscle in D2.*mdx* mice up to 70 days following a single injection. Furthermore, myofiber size and TA muscle weight were greater following CoO-TRIM-treatment, which improved max isometric force and reversed atrophy without any changes in TA muscle fiber type (Supplementary Figure 3).

In association with the enhancement of muscle strength and myofiber size, we investigated activation of the Akt-mTOR pathway and its downstream target, 4E-BP1. Remarkably, CoO-TRIM did not restore muscle function through increased muscle anabolism, as key markers of anabolism were not elevated (Figure 7). Alternatively, increases in muscle function and structure may be explained by suppressed proteolytic activity. Calpains initiate widespread proteolysis, contributing to muscle cell death and DMD pathology^74,98,99^. Our results following TRIM injection trended towards reduced calpain activity at 14 dpt, and a significant decrease at 70 dpt (Figure 8A and 8B). These findings coincide with previous reports that calpain inhibition in *mdx* mice increases myofiber size and function^75,100^. In addition, CoO-TRIM reduced autophagosome protein marker LC3II^101^ (Figure 8D and 8F). Therefore, we conclude that CoO-TRIM restores muscle size and contractile force by reducing proteolytic activity, thereby enhancing myofiber regeneration.

### CoO-TRIM promotes VEGF elevation and angiogenesis

Vascular dysfunction is a prominent contributor to DMD marked by a reduction in endothelial cell density, impaired angiogenesis, perturbations in vasodilation, and insufficient blood supply to degenerating muscles^18,102–104^. Reduced muscle damage in dystrophic mice is associated with enhanced angiogenesis with overexpression of VEGF^21,27–29^. The ions released from CoO-TRIM stimulate VEGF production and subsequent angiogenesis^39,67,78,88^, with cobalt acting as a hypoxia mimetic through binding to HIF-1α and inducing its translocation to the nucleus, thereby activating VEGF release^39,68,90,105^. Regarding the use of VEGFA as a therapeutic agent, its short half-life (<30 minutes) and its off-target effects have prevented the use of VEGF in the clinic. As an alternative strategy for inducing long-term enhancements to VEGF, the present findings reveal the safety and efficacy of CoO-TRIM.

Duchenne Muscular Dystrophy presents additional challenges for systemic therapies. As *DMD* gene mutations reduce the activity of neuronal nitric oxide synthase (nNOS)^106^, questions arise regarding the functional nature of angiogenesis in DMD. However, early studies have inhibited nNOS activity during exercise demonstrating no differences in muscle blood flow suggesting nNOS is not required for functional hyperemia^107,108^. The current investigations evaluated perfusion in TRIM treated D2.*mdx* mice with intravascular WGA used to stain the endothelial cell glycocalyx. Therefore, not only are there more microvessels within dystrophic skeletal muscle following TRIM treatment, but the increase in perfusion also enables more effective delivery of systemic therapeutics. Despite an increase in vascularity and perfusion, there did not appear to be an increase in muscle mitochondrial volume (Supplementary Figure 4).

The present study encompassed 3 timepoints. Concomitant with increased VEGF in the TA of TRIM-treated *D2.mdx* mice, muscle function, size, and vascular area were improved (14 and 70 dpt). At 140 dpt, while vascular area was enhanced, VEGF was no longer elevated. At this later time point, max isometric force and myofiber CSA were similar to *D2.mdx* Saline. These findings are the first to uncouple VEGF mediated enhancement of muscle function in *mdx* mice from angiogenesis. In particular, at 140 dpt, angiogenesis was elevated while VEGF was no longer different from *D2.mdx* Saline, nor were max force or myofiber CSA. Therefore, VEGF promotes enhancements to dystrophic muscle function and CSA in addition to angiogenesis. In that regard, VEGF promotes proliferation and maturation of satellite cells that may contribute to the replenishment of the impaired regenerative capacity seen in DMD^30,32,109,110^, enhancing dystrophic skeletal muscle structure and function.

## 5. Conclusion

Ion matrices have been used to stimulate regeneration in the context of bone growth, wound healing, and volumetric muscle loss. Herein, we show that TRIMs serve as effective therapeutics to stimulate angiogenesis and myofiber regeneration in the context of treating MD. The present findings in dystrophic mouse skeletal muscle suggest CoO-TRIM’s ion release from a single treatment stimulates VEGF availability as early as 14 dpt and as long as 70 dpt. These results support our hypothesis that CoO-TRIM can enhance muscle force and myofiber CSA in skeletal muscle, as shown in D2.*mdx* mice. Furthermore, increased growth factors promoted angiogenesis and increased microvascular area associated with myofibers. We conclude that CoO-TRIM induces complementary effects on angiogenesis and muscle regeneration capable of restoring dystrophic muscle function.

## 6. Translational Perspective

Duchenne Muscular Dystrophy is a debilitating disease, leading to loss of skeletal muscle function in adolescence, increasing the burden upon caregivers. While multiple therapeutics have been developed to reduce the severity and progression of DMD, few are applicable to patients of all ages without side effects. Furthermore, there is minimal recovery of muscle mass, function, or growth of healthy blood vessels in otherwise ischemic and/or necrotic muscles using current treatments. CoO-TRIM is an injectable biomaterial undergoing late-stage preclinical trials as an FDA designated Orphan and Rare Pediatric Disease Drug, releasing therapeutic ions over time in an injected myofascial compartment. The goal is to use CoO-TRIM to enhance muscle function and vascular supply in human males with DMD. In hindlimb muscles of severely dystrophic mice (>6 mo old D2.*mdx*), a single injection of CoO-TRIM enhanced muscle function within 14 dpt and benefits lasted for as long as 70 dpt. Increases in function corresponded to a reduction of small myofibers and an increase in large myofibers accompanied by elevated VEGF through 70 dpt. Counteracting ischemia within muscles affected by DMD, CoO-TRIM enhanced vascular area at all time points. We suggest that CoO-TRIM acted as a hypoxia mimetic, thereby providing a clinically relevant method for promoting sustained, local VEGF production without off-target effects. CoO-TRIM is delivered as a lyophilized powder contained within a vial, resuspended with saline in the clinic prior to injection within affected myofascial compartments. A single injection enhances muscle function of murine dystrophic muscle for the short and midterm while promoting a sustained vascular supply for more effective delivery of systemic drugs, oxygen, nutrients, and removal of metabolic by-products in severely affected muscles.

## Figures and Tables

**Figure 9.**
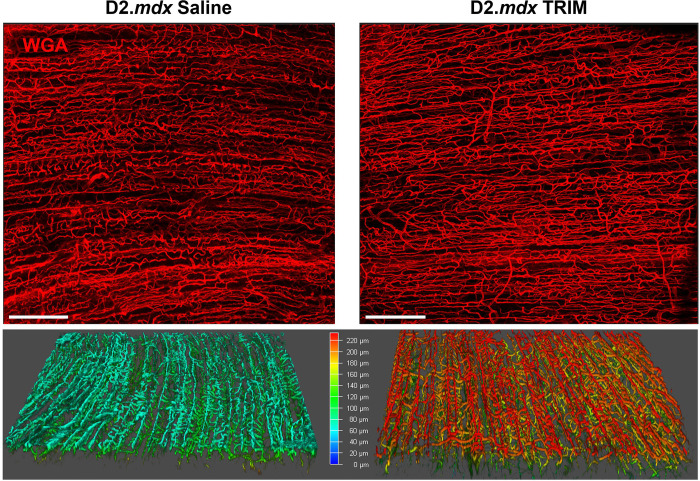

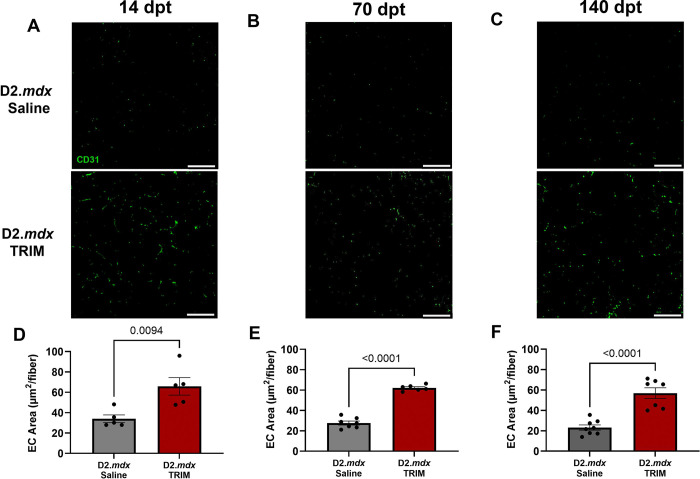
TRIM enhances angiogenesis. **9.1,** Capillary networks stained *en face* with intraluminal dye (WGA) in the GM of D2.*mdx* mice without and with CoO-TRIM 14 dpt. Top) TRIM appeared to show increased capillary diameter and Bottom) greater depth of vessel perfusion was detected indicating robust capillary networks in TRIM treated muscles compared to untreated. Colors in lower, rotated panels: Cyan represents reduced vascular density; Red indicates greater vascular density. Scale bars = 200 μm. **9.2, A-C)** Representative images of TA cross-sections in Control and TRIM mice at **A)** 14 dpt, **B)** 70 dpt, and **C)** 140 dpt. CD31+ (green) identifies endothelial cells as a measure of angiogenesis. Summary values for microvascular area was calculated as; total endothelial cell area/ # of myofibers. Summary values presented for **D)** 14 dpt, **E)** 70 dpt, and **F)** 140 dpt. (n=5–8); Summary values are means ± SEM, p<0.05 = significant. Scale bars = 100 μm.

**Table 1.** Body weight, TA muscle weight and peak isometric force of D2.*mdx* mice at 14−, 70−, and 140 dpt.

	14 dpt		70 dpt		140 dpt	
	D2.*mdx* Saline	D2.*mdx* TRIM	D2.*mdx* Saline	D2.*mdx* TRIM	D2.*mdx* Saline	D2.*mdx* TRIM
**Body Weight (g)**	27.75 (1.07)	28.61 (0.96)	25.89 (0.43)	27.60 (1.15)	24.98 (0.44)	**27.22 (0.66)** *
**TA Weight (mg)**	29.00 (1.27)	31.29 (1.27)	31.97 (1.05)	34.14 (1.09)	30.61 (1.39)	35.52 (1.88)
**Max Direct Force (g)**	55.21 (2.15)	**64.69 (3.14)** *	65.86 (1.11)	**70.86 (1.81)** *	56.34 (1.51)	59.24 (2.67)
**Max Direct Force (N/cm^2^)**	26.73 (2.11)	27.76 (0.76)	30.00 (0.80)	29.56 (1.02)	26.15 (0.59)	23.33 (2.20)
**Max Indirect Force (g)**	46.10 (2.63)	**56.61 (3.40)** *	60.79 (1.85)	66.08 (1.71)	52 98 (1.36)	52.76 (3.56)
**Max Indirect Force (N/cm^2^)**	22.50 (2.34)	24.25 (0.94)	27.65 (0.81)	27.57 (1.00)	24.75 (0.66)	21.55 (2.16)
**Direct-Indirect Force (g)**	9.10 (1.76)	8.08 (1.23)	5.08 (1.12)	4.78 (0.49)	3.36 (0.39)	**5.08 (0.45)** *

Summary values are means (SEM), *p<0.05 = significant.

## Data Availability

The data that support the findings of this study are available from the corresponding author upon reasonable request.
